# Case of Peripneumonia, Which Terminated Fatally in Arteritis: With Observations and Dissections of Other Cases

**Published:** 1821-12

**Authors:** C. W. Smerdon

**Affiliations:** Surgeon.


### Original Communications, Select Observations, etc.

*Case of Peripneumonia, which terminated fatally in Arteritis: with Observations and Dissections of other Cases.* By C. W. Smerdon, Surgeon.

THE subject of the following case and observations was a coachman, aged 38, of a strong muscular make, inclined to corpulency, with a short thick neck, and florid complexion. He has had four severe attacks of pneumonia within the last two years, in each of which the most active depletion was absolutely required for his recovery. The last of these attacks was more severe than the preceding ones ; and his respiration afterwards remained permanently oppressed, attended with a hoarse phthisical cough. About three months after this imperfect recovery, namely, on the 11th of April, 1818, he again became the subject of the same disease. This attack was ushered in by a severe febrile paroxysm ; after which his respiration becan e extremely laborious, and was attended by a hard dry cough, and severe pains of the chest and left hypochondrium, shooting to the spine. His face was flushed, and the vessels of the conjunctiva were turgid with red blood. His pulse was vacillating and weak. An emetic was prescribed, and a large dose of calomel and antimonial powder, with salts for the morning. The report of the next day is as follows :—His pulse, this morning, is full, and bounds under the pressure of the finger, at 120 beats in the minute. Tongue coated ; respiration extremely laborious; he expectorates, with convulsive efforts, a little frothy mucus ; but his cough, for the most part, is hard and dry. His skin is hot and arid ; countenance depressed, and face of a purple redness. He complains of severe pains in the loins, chest, between the shoulders, and left hypochondrium. The medicines have acted powerfully on his bowels.—Mittatur sanguis ad deliquium animi. Rep. remedia purgantia.

From this time to the 17th inclusive, the violence of the disease continued with but little abatement, notwithstanding the following vigorous means had been resorted to:—He was bled to two pounds, on the 13th; on the 14th, to twenty-four ounces, and a blister applied to the chest; on the 16th, he was again bled to two pounds, with a blister between the shoulders; and, on the 17th, he was bled to twenty-four ounces. Each night, from the commencement of the disease, he had taken calomel, gr. vj. and pulv. antimon. gr. iv., with salts in the morning. On the 18th, he appeared to be considerably better. The pains, which he complained of before, were much mitigated; the pulse was reduced both in frequency and strength; and he expectorated with much greater freedom. He was still restrained to a vegetable and farinaceous diet; and ext. hyoscyamus and ext. coloc. comp. āā gr. v. were ordered for him every night.

Up to the 21st, he appeared to have been gradually improving, but on that morning the disease again assumed a menacing aspect, the report of which is as follows:—He complains of soreness of the throat, and pressure below the thyroid cartilage gives him uneasiness. He swallows with some difficulty; and, what appears very remarkable, can only speak in a whisper. His respiration is laborious, especially when he lies down in a directly horizontal line. He has a hoarse, and rather dry, cough; his appetite is good; tongue brown towards the back part, but cleaner at its tip. He has more difficulty in expelling than in inhaling a full inspiration: it appears as if there were imperfect valves in the bronchial tubes, which impeded the passage of the air outwards. He has no pain, but complains much of a sense of fullness in the left hypochondrium. Bowels freely open; pulse denoting power at 110 in the minute, but not full.—Calomel, with Dover's powders, was ordered in small doses three times a-day.

With a view to ascertain the cause of the fullness of which he complained, I examined the left hypochondrium. A strong pulsation, synchronous with the artery at the wrist, was felt a little to the left of the scrobiculus cordis. This pulsation the patient distinctly felt when pressure was made over the part, and respiration was then particularly distressing to him.

On the 22d, a blister was applied to the chest, which gave him but temporary relief. On the 24th, he again became much worse. Pulse, 120 beats in the minute, tolerably strong; voice scarcely audible, and the pain on swallowing much increased. Pressure about the middle of the trachea gave him much uneasiness: he complained likewise of pain between the shoulders, and of the fullness in the left hypochondrium; the pulsation in the part was still the same. At noon he was seized with a convulsive paroxysm, or with an attack resembling apoplexy. He died the next morning.

*Body examined fourteen hours after death.—Thorax.*—The viscera, *in situ*, with their investing membrane, exhibited a much paler appearance than what is natural to them. The lung of the left side partially adhered to the pleura costalis, and a pint and half of bloody serum was found in this cavity. After a careful examination, not a trace of disease could be discovered within the pharynx, larynx, or trachea; but, on lifting up the lung, a quantity of thin, colourless fluid was thrown up, and with this the bronchial ramifications were found, on examination, to be filled. The substance of the lung was so soft as to be easily crushed by the gentlest pressure of the hand; and, by this means alone, full six ounces of fluid were squeezed out. The lung on the opposite side was affected by the same disease, although in a much less degree. The pericardium contained about an ounce of fluid. The heart was very flaccid, and its appearance unusually pale. With the view of examining this viscus at home, I removed it from the body; taking care to preserve portions of the large vessels along with it.

*Abdomen.*—The viscera in this cavity, with the exception of the spleen, were healthy: the substance of that viscus was very soft, and appeared disorganized.

The heart was small in proportion to the size of the subject from whom it was taken; the parietes of its cavities were thin, and the muscular structure exhibited a pale leaden-coloured appearance. The membrane lining the internal surface of the aorta exhibited marks of the most extensive inflammation: it was much thickened and hard, and was easily stripped off, even with the nail. The surface, commencing from the semilunar valves, was red throughout the whole of the parts that were saved,—namely, as far as the curvature, a small portion of the arteria innominata, and left carotid. Large elevated patches of the membrane, more thickened than the rest, were seen here and there, of a deep crimson colour. In the pulmonary artery, the inner coat was natural; and this, when contrasted with the morbid structure of the aorta, made the disease appear still more striking.

This case was evidently peripneumonia, which terminated fatally in arteritis; but there are some circumstances connected with it, on which a few observations may profitably be made. I have said "terminated fatally in arteritis," because we have no proof of the existence of the latter affection until after the pneumonic symptoms had given way, and when, indeed, the patient had been, for some days, considered in a fair way of doing well. On the 21st, new (and, I may say, obscure) symptoms made their appearance; and from this period, I think, we may reasonably date the commencement of the arterial inflammation.

The pulsation in the epigastric region, which made such a prominent feature in the latter progress of this disease, was symptomatic of the arterial inflammation. In the Medico-Chirurgical Review for June last, there is a well-written paper on the symptoms of arteritis, among which the following are mentioned:—The lips are crimson or livid; tongue red, smooth, moist; at the same time, the breathing is rapid, difficult, and irregular. Pulse hard, labouring, and accelerated to 110—130; a distressing sensation of constriction is established, most frequently in the cardiac, præcordial, or epigastric, region; forcible palpitation of the heart, and, in some instances, of the abdominal aorta, so as to be distinctly felt through the abdominal parietes, now predominate."

---

The following *post mortem* appearances of another case of arteritis, which terminated in ulceration of the aorta, and consequent extravasation of blood into the pericardium, I will take leave to note down here.

G. B. Esq. aged 57, died suddenly in the night of the 4th of October, 1817. He had long been the subject of chest-complaint, which was supposed to arise from a disease of the heart. As I did not see this gentleman during his life-time, I regret much that I am unable to state the symptoms which marked the progress of this very interesting case.

*Thorax.*—The left lung was diseased in consequence of former inflammation: it was gorged with bloody serum, which oozed out after each stroke of the knife. The right lung was healthy, and perfectly free from adhesions; but, in the cavity of this side of the chest, there were found about fourteen or fifteen ounces of serum slightly tinged with blood. The pericardium was greatly distended with fluid. A firm coagulum of very black blood, amounting to about twelve ounces, was found within it; together with a pint, or upwards, of bloody serum. The common membrane which surrounds the aorta only adhered to it by means of slightly elongated cellular tissue, and the small interstices, thus formed, were filled with black coagulated blood. This morbid structure extended throughout the whole of the thoracic portion of the vessel, (beyond which its examination was not carried,) and appeared also to pervade the branches which go off from the curvature. In this membrane, or (more correctly) in that portion of the pericardium which closely invests the aorta, a rupture was found, about a quarter of an inch above the base of the heart; and, a few lines below, another was discovered in the aorta itself, of which no part was in apposition with the external opening. The rupture of the aorta was seated just above the semilunar valves, and was large enough to admit the top of the finger. At this part the coats of the vessel were very thin, but no appearance of dilatation could be perceived, which is so commonly found in aneurisms of the aorta. The internal surface of the vessel was very thin and irregular, being much puckered up and striated, and thickly studded with yellowish spots of different sizes, which appeared to be the consequence of an ulceration of its inner coat. The irregularity of surface was most conspicuous beyond the curvature, occasioned by a number of small scrofulous tumors containing cheesy matter, and which were seated between the elastic and the muscular coat. The coats of the vessel were easily detached from each other, and these again were very readily separated into layers.

In the fourth volume of the Medical Transactions of the College of Physicians, there is a paper, by Dr. Baillie, on Pulsation in the Epigastric Region; in which that distinguished physician discountenances the opinion that this affection is frequently the consequence of diseased structure, but rather the effect of indigestion, or of loaded or obstructed bowels.

That a pulsation in the epigastric region may be the effect of indigestion, without supposing the existence of any organic disease, I am well aware of, from my own feelings. Frequently after having eaten some heavy food, particularly pastry at dinner, I have the sensation of a strong pulse a little below the scrobiculus cordis, inclining to the left side. As far as I can judge, the aorta, where it gives off the cœliac artery, is the part affected. Pressure over it with the hand gives considerable uneasiness; the pulsation being thereby rendered stronger, as well as very apparent to the feel, and sometimes likewise to the eye. It is usually accompanied with fullness in the epigastrium; eructations of flatus; an unpleasant clamminess in the mouth; an uneasy, dull sensation of weight and fullness over the eyes, and along the forehead, which is hotter than natural; noise in the ears; disposition to frequent deep inspirations; languor, and lowness of the spirits; an increased quantity of pale, and almost inodorous, urine. These unpleasant symptoms of indigestion are never present but when the bowels are constipated; and, consequently, are relieved when the cause is removed by mild aperients. I should have stated, that the epigastric pulsation is seldom felt but when in bed. The sleep which follows is much disturbed by dreams.

Another case of pulsation in the epigastric region I met with some time back: the subject of it was a woman, who died of inanition in consequence of a stricture of the pylorus. In this case the pulsation was very strong, and there was a hard lobulated tumor in the part where the pulsation was felt. On examination, the stomach presented itself very much enlarged at the cardiac extremity: here the parietes of the viscus were very thin, apparently the consequence of a long and gradual distension; but, from the middle of the lesser curvature extending to the pylorus, they were preternaturally thickened, which gradually increased as we approached the pylorus, the aperture of which was unyielding and nearly closed. The little omentum was converted into a large scirrhous-like mass; being hard, puckered up, and considerably thickened. The peritoneum covering the pancreas was converted into the same diseased structure, and the head of the pancreas was also hard and lobulated. In this case, the epigastric pulsation was evidently occasioned by the pressure of the hardened viscera on the abdominal aorta or its branches. The large vessels were not examined; an omission which I should not be guilty of again, if a similar opportunity should occur.

The loss of voice and soreness of the throat, noted in the coachman's case, are singular instances of sympathetic influence: nor do I know to what cause they are referrible, unless the arterial inflammation acting, through the medium of the recurrents, on the larynx and trachea, may be considered as such. The singular course of the recurrent nerves is too well known for me to describe. By their distribution to the larynx, and the junction of some of their branches with the pulmonary plexusses, they have justly been considered as the nerves of voice: why they should wind round the subclavian artery and the arch of the aorta to reach their principal destination, must rank among the many obscurities in the distributions of nerves which we have yet to learn; but, if the above inference be allowed, that the loss of voice was the consequence of the diseased state of the aorta and its branches, then the natural conclusion will be, that there is an intimate, and no doubt a necessary, connexion between the large blood-vessels of the thorax and the vocal organs; and that the origin and peculiar course of the recurrent nerves are contrivances of nature for that purpose. On this subject, Dr. Barclay, after describing the course of the recurrent nerves, thus concludes:—"In consequence of this singular course, when the action of the heart or arteries is changed, we generally find that a part of the change is indicated by the voice."—*On Vascular Motion*, p. 254.

A lady, who was for many years an invalid, and whose disease throughout was very obscure, during the progress of it was suddenly attacked with loss of voice and an intermitting pulse. This intermission appeared to be dependant on a like affection of the heart, which she was quite sensible of. A blister to the chest gave her complete, though but temporary, relief. In the evening, the loss of voice recurred again, and, what is singular, the intermission also; proving that they were dependent on one and the same cause. Bleeding, and a few calomel purges, were the only means that gave her permanent relief; but, from the several attacks which she had of this singular affection, it would seem that, whenever her constitution required powerful remedies of that kind, it was indicated by an intermission of the pulse and loss of voice.

I am unwilling to expatiate further on this subject. What I have written will not, I trust, prove wholly uninteresting. The laws of sympathy are very obscure; and he who contributes his mite towards their elucidation will, I am sure, render some service to the healing art.

*Clifton, Bristol; Oct.* 9, 1821.

